# 

**Published:** 1815-02

**Authors:** 


### MONTHLY CATALOGUE OF MEDICAL BOOKS.

THE Physiognomical System of Drs. Gall and Spurzheim, founded on an Anatomical and Physiological Examination of the Nervous System in general, and of the Brain in particular, and indicating the Dispositions and Manifestations of the Mind. By J. G. Spurzheim, M.D. Illustrated with nineteen copper plates. Royal 8vo.—Baldwin and Co.

Delineations of the Cutaneous Diseases comprised in the Classification of the late Dr. Willan: being a re-publication of the greater part of the Engravings of that Author, in an improved State, together with a new Series, which will comprehend the remainder of the System, as completed in the Practical Synopsis of Dr. Bateman; the whole being intended to illustrate the principal Genera and Species described in that Work. By T. Bateman, M.D. F.L.S. Fasciculus 1st. 4to.—Longman and Co.

Observations on the Animal Economy. By a Physician.—Callow.

An Illustration of Mr. Hunter's Doctrine, particularly concerning the Life of the Blood, in Answer to the Edinburgh Review of Mr. Abernethy's Lectures. By Joseph Adams, M.D. 8vo. Callow.

The Case of Joanna Southcott, as far as it came under his Professional Observation, impartially stated. By P. Mathias.—Callow.

The Surgeon's Vade-Mecum, containing the Symptoms, Causes, Diagnosis, Prognosis, and Treatment, of Surgical Diseases, accompanied by Engravings, to illustrate the modern and approved Methods of Operating; also, select Formula of Prescriptions, and a Glossary of Terms. 12mo.—Longman and Co.

The Art of Preserving the Sight unimpaired to an extreme Old Age, and of re-establishing and strengthening it when it becomes Weak; to which are added, Observations on the Inconveniences and Dangers arising from the use of common Spectacles, &c. &c. By an experienced Oculist. Second edition. 12mo.—Colburn.

The Institutions of Physiology, translated from the Latin of Professor Blumenbach, with additional Notes, illustrative and emendatory. 8vo. boards.—Cox and Son.

A Bill for Enlarging the Charter of the Society of Apothecaries in the City of London, granted by his Majesty King James the First, and for better Regulating the Practice of Apothecaries throughout England and Wales. 8vo. sewed.—Callow.

Anatomical Plates of the Bones and Muscles, diminished, from Albinus, for the use of Students in Anatomy and Artists, accompanied with explanatory Maps. By Robert Hooper, M.D. A new Edition, in 12mo.—Callow.

Anatomical Plates of the Thoracic and Abdominal Viscera, for the use of Students in Anatomy and Artists, accompanied by explanatory Maps. By R. Hooper, M.D. A new ed. 12mo.—Callow.

---

### TO CORRESPONDENTS.

*We regret that Mr. Stephenson's Communication arrived too late for the present month. We have also been favoured, as usual, with several Articles, which want of room, and the lateness with which some of them were received, have occasioned us to defer till a future Number. Among them are Communications from Drs. Clarke, Robertson; Messrs. Hey, Bush, Walker, Rowe, Muchell, &c. &c.*

